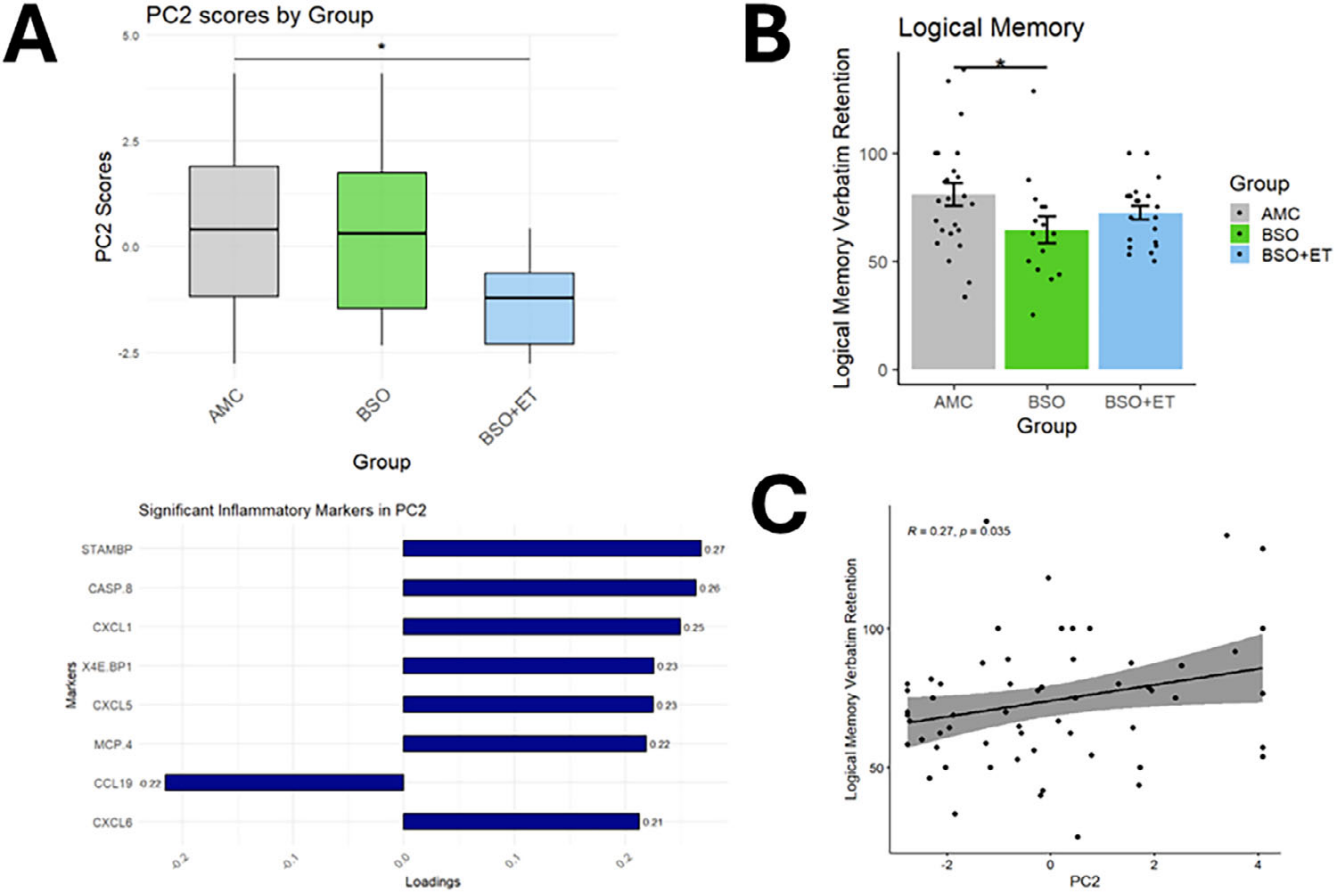# Inflammation and verbal episodic memory correlate with bilateral salpingo‐oophorectomy in early midlife

**DOI:** 10.1002/alz70861_108904

**Published:** 2025-12-23

**Authors:** Sophia Zhao, Alana Brown, Laura Gravelsins, Shreeyaa Ramana, Noelia Calvo, Emily Pullen, Elisabet Classon, Elvar Theodorsson, Preben Kjølhede, Shannon Dunn, Gillian Einstein

**Affiliations:** ^1^ University of Toronto, Toronto, ON Canada; ^2^ Rotman Research Institute, Baycrest Academy for Research and Education, Toronto, ON Canada; ^3^ Centre for Addiction and Mental Health, Campbell Family Mental Health Research Institute, Toronto, ON Canada; ^4^ Sunnybrook Research Institute, Toronto, ON Canada; ^5^ Linköping University, Linköping Sweden; ^6^ Women's College Research Institute, Toronto, ON Canada; ^7^ Linkoping University, Linkoping Sweden; ^8^ Rotman Research Institute, Toronto, ON Canada

## Abstract

**Background:**

Women are disproportionately affected by Alzheimer’s disease (AD), and 17β‐estradiol loss is related to pathologies of AD including heightened systemic inflammation and reduced performance on verbal episodic memory tasks (Tani et al., 2013; Gervais et al., 2020). Women with bilateral salpingo‐oophorectomy (BSO; removal of ovaries and fallopian tubes) experience an early and abrupt loss of 17β‐estradiol, leading to increased risk of late‐life AD compared to women with intact ovaries (Rocca et al., 2007; Calvo et al., 2024). The purpose of this study was to investigate whether systemic inflammation develops after BSO and if it associates with cognitive changes.

**Method:**

Participants included women with BSO taking estradiol therapy (BSO+ET; *M±SD_age_* =44.3±6.0, *n*=22) or not taking ET (BSO; *M±SD_age_* =47.3±5.9, *n*=16), and age‐matched premenopausal controls (AMC; *M±SD_age_* =43.5±3.3, *n*=26). Using the OLINK platform, 92 proteins were measured, and dimensionality reduction was performed using Principal Component Analysis (PCA). Verbal episodic memory was measured using verbatim retention in the logical memory task; group differences were determined using a linear regression. Inflammatory factors identified by PCA were compared amongst groups and correlated with verbatim retention scores.

**Result:**

The first two principal components (PC1 and PC2) explained 30.5% and 11% of the variance in protein marker expression, respectively. There were no group differences in PC1 score. However, group comparisons revealed that PC2 score was lower for BSO+ET compared to AMC; PC2 included neutrophil‐activating chemokines (CXCL1, CXCL5, CXCL6) and monocytes (MCP‐4) (Figure 1A). Women with BSO had significantly lower verbatim retention compared to AMC, while ET partially alleviated this effect of BSO (Figure 1B). PC2 score associated weakly with retention scores (Figure 1C).

**Conclusion:**

Women with early BSO both taking and not taking ET showed no significant elevations in inflammatory protein markers, suggesting an absence of chronic systemic inflammation in early midlife. ET correlated with reduced chemokine expression and reduced neutrophil and monocyte recruitment systemically, suggesting anti‐inflammatory mechanisms. Other known markers associated with cognitive decline, including c‐reactive protein (CRP), were not included in the panel, however this study widens the scope of inflammatory markers studied. The correlation between reduced PC2 and verbal retention suggests a possible role of inflammation in cognition.